# Apple Seed Extract in Cancer Treatment: Assessing Its Effects on Liver Damage and Recovery

**DOI:** 10.3390/cimb48010055

**Published:** 2026-01-01

**Authors:** Min-Jee Oh, Yong-Su Park, Ji-Yeon Mo, Sang-Hwan Kim

**Affiliations:** 1 General Graduate School of Animal Life Convergence Science, Hankyong National University, 327, Jungang-ro, Ansung 17579, Gyeonggi-do, Republic of Korea; wertey08@naver.com (M.-J.O.); mjy010627@naver.com (J.-Y.M.); 2 Institute of Applied Humanimal Science, Hankyong National University, 327, Jungang-ro, Ansung 17579, Gyeonggi-do, Republic of Korea; 3 Research Center for Endangered Species, National Institute of Ecology, 1210, Geumgang-ro, Maseo-myeon, Seocheon-gun 33657, Chungcheongnam-do, Republic of Korea; muskdeer@nie.re.kr; 4 School of Animal Life Convergence Science, Hankyong National University, 327, Jungang-ro, Ansung 17579, Gyeonggi-do, Republic of Korea

**Keywords:** apple seed extract, amygdalin, apoptosis, mouse, liver

## Abstract

Cancer therapies frequently induce hepatotoxicity, complicating treatment courses and outcomes. Natural products, including polyphenol-rich extracts, have shown hepatoprotective activity via anti-oxidative and anti-inflammatory mechanisms, often linked to NF-κB and PI3K–Akt pathways. Apple-derived polyphenols (e.g., phlorizin/phloretin) also demonstrate liver-protective effects in experimental settings. In this study, we examined whether ASE mitigates cancer-related liver damage by rebalancing the apoptosis–survival axis and maintaining PI3K-Akt signaling in an endometrial cancer mouse model. Female Institute of Cancer Research mice with induced endometrial cancer received ASE (0–200 mg) over 13 days; liver tissues were analyzed for Caspase-3, p53, LC3, and SQSTM1 using histology stains, Western blot (e.g., Caspase-3/9, Bcl-xL, PI3K, Akt, PCNA, IGF-IR), ELISA, and qRT-PCR (GAPDH). ImageJ (version 1.54f; RRID: SCR_003070) quantification statistical analysis followed (mean ± SD; post-hoc tests). ASE exhibited dose-dependent modulation of apoptosis and survival readouts in liver tissue of cancer-bearing mice: (i) Caspase-9/3 and Bcl-xL showed differential regulation across doses; (ii) PI3K–Akt and IL-2 signals were preserved or restored toward baseline at specific doses; and (iii) histology indicated partial structural recovery. Thus, ASE may mitigate liver injury by re-balancing apoptosis–survival signaling and promoting structural recovery. Our interpretation emphasizes that dose, route, and formulation are critical for translational potential.

## 1. Introduction

Antineoplastic therapy frequently precipitates hepatotoxicity, spanning drug-induced liver injury, cholestasis, and sinusoidal obstruction. Hepatic adverse events often drive dose modification, treatment interruption, and poorer overall outcomes [1]. At the molecular level, such injury reflects the intersecting axes of oxidative stress, inflammation, and cell death regulation, with the recurrent involvement of NF-κB, Nrf2/HO-1, and PI3K–Akt signaling across experimental and clinical contexts [2].

In endometrial cancer, the liver is a clinically important site of distant spread. Population-based analyses have indicated that liver metastasis, although less frequent than lung involvement, is an adverse prognostic factor for overall and cancer-specific survival [3]. These observations underscore the need for hepatoprotective strategies for cancer-associated liver injury in which redox-inflammatory and survival pathways are dysregulated.

Natural product approaches for hepatoprotection continue to accrue preclinical support [4]. Among these, apple-derived polyphenols (e.g., phlorizin and phloretin) have been shown to improve serum transaminase levels and histopathology in toxin-induced animal models, and have been mechanistically linked to the restoration of antioxidant defenses and modulation of survival signaling [5,6]. For example, apple polyphenol extract mitigates aluminum-induced hepatic oxidative stress in rats by attenuating the oxidative burden and improving biochemical indices [5]. Conceptually, these data provide a rationale for testing whether apple-origin bioactives can rebalance apoptosis–survival nodes and preserve PI3K–Akt activity under oncological conditions that impose hepatic stress.

A key safety consideration is that apple seeds contain amygdalin (Laetrile) which metabolizes into cyanide, a fatal toxin. Authoritative summaries from the National Cancer Institute Physician Data Query (PDQ) conclude that anticancer activity of apple-origin bioactives is not supported in human trials owing to documented cyanide-consistent toxicities [7]. Nevertheless, some animal studies have reported that amygdalin improves the alanine aminotransferase/aspartate aminotransferase (ALT/AST) ratio and pathology in LPS/D-GalN- or acetaminophen-induced acute liver injury models [3,8]. Any evaluation of apple seed extract (ASE) should, therefore, be anchored in a defined dose, formulation, and route, with objective safety biomarkers (e.g., ALT/AST) assessed alongside efficacy.

Such profiling will also allow determination of ingredient-level dose ranges that best balance potential cancer cell-killing activity with hepatoprotective efficacy.

Although research on ASE remains limited, one report in a mouse endometrial-cancer model describes modulation of the TNF-α/p53 axis, reduced NF-κB expression, and changes in apoptosis-related markers [9]. Building on these observations and on the polyphenol literature, we hypothesized that ASE could mitigate cancer-associated liver injury by constraining oxidative/NF-κB-driven inflammation, preserving PI3K–Akt survival signaling, and thereby limiting mitochondrial caspase-9/3 activation. Key proteins of the autophagy pathway, LC3/SQSTM1, were included as autophagy readouts to contextualize molecular changes with tissue remodeling [2,4,5].

Among apple-derived preparations, this study focused on apple seed extract (ASE). Apple seeds are rich in polyphenols, a less well-studied ingredient, and also contain cyanogenic compounds such as amygdalin. This apple seed matrix allowed us to investigate the effects of this compound on cancer-related liver damage in a setting where both potentially beneficial and toxic compounds exist. This approach contrasts with pulp or peel extracts, which contain little cyanogenic compounds.

Therefore, given the clinical relevance of hepatic involvement in endometrial cancer, this study analyzed the effects of ASE on liver tissue in a mouse endometrial cancer model to determine whether ASE confers beneficial effects on tissue restructuring and physiological functions [3]. Accordingly, we tested whether ASE attenuates cancer-associated hepatic injury by rebalancing the apoptosis–survival axis and preserving PI3K–Akt signaling in an endometrial cancer mouse model.

## 2. Materials and Methods

### 2.1. Extraction and Concentration of Apple Seed Ethanol Extract

The apple seeds were dried and crushed. They were extracted into 80% ethanol by Rotary evaporator (Eyela N-1300V-W, Tokyo, Japan) for 48 h at 50 °C and 100 RPM. The collected extract was concentrated to 15 g/50 mL at 45 °C and 100 RPM. ASE, amygdalin, and phlorizin were dissolved in 0.1% ethanol, and the same final concentration of ethanol was applied to the vehicle control group.

### 2.2. Primary Hepatocyte Isolation and Culture

Primary hepatocytes were isolated from 12-week-old female Institute of Cancer Research (ICR) mice under a protocol approved by the Institutional Animal Care and Use Committee (HK-2022-4) of Hankyong National University. Liver tissues were excised and washed with Dulbecco’s phosphate-buffered saline (D-PBS), and then incubated in 0.2% Trypsin-EDTA at 37 °C for 1 h. The resulting cell suspension was filtered through a 70 µm cell strainer and centrifuged at 1500 rpm for 5 min. Cells were resuspended in Dulbecco’s Modified Eagle Medium (DMEM) supplemented with 10% fetal bovine serum and 1% antibiotic–antimycotic solution and seeded into culture plates at a density of 1 × 10^6^ cells per well.

The dose ranges for amygdalin and amygdalin plus phlorizin were determined based on previous research [3]. For ASE toxicity assessment, primary hepatocytes were treated with ASE at concentrations ranging from 0 to 100 µg/mL for 48 h, and cell viability was measured by CCK-8 assay. Cell viability data were fitted to a sigmoidal dose–response curve, and the IC50 value of ASE (74.7 µg/mL) was estimated from the fitted dose–response curve using Microsoft Excel 2019 (Microsoft Corp., Redmond, WA, USA). Based on these results, three ASE concentrations (20, 30, and 40 µg/mL), together with the corresponding amygdalin and amygdalin plus phlorizin doses (20, 30, and 40 µM), were selected for subsequent experiments. Amygdalin (A) and amygdalin plus phlorizin (AP) groups were therefore treated at 20, 30, or 40 µM for 48 h, and ASE was applied at 20, 30, or 40 µg/mL for 48 h.

### 2.3. Cell Survival Assay

Cell survival assay was analyzed in BD Pharmingen FITC Annexin V Apoptosis Detection Kit I (BD Biosciences, Franklin Lakes, NJ, USA). The treated cells were washed twice with cold phosphate-buffered saline (PBS) and resuspended cells in 1 × Binding Buffer at a concentration of 1 × 10^6^ cells/mL. Subsequently, 100 µL of the solution (1 × 10^5^ cells) was transferred to a 5 mL conical tube and 5 µL of Annexin V and 5 µL PI was added to it. The cells were gently vortexed and incubated in the dark for 15 min at room temperature. About 400 µL of 1 × binding buffer was added to each tube. Analysis was conducted using flow cytometry within 1 h.

### 2.4. Cancer Mouse Model

All experiments were performed according to a protocol approved by the Institutional Animal Care and Use Committee (IACUC 2022-4) of Hankyong National University. Female ICR mice were purchased from Nara-Biotec (Pyeongtaek, Republic of Korea) and housed individually under controlled conditions (25 °C, 50% relative humidity, 12 h light/dark cycle; Type III cages, 420 mm × 260 mm × 150 mm).

Endometrial cancer-like lesions were induced using a protocol adapted from Kim et al. [9]. Briefly, 1 × 10^6^ HCT116 (human colorectal carcinoma cells) suspended in 100 µL of 1× PBS were injected into the lower abdominal cavity along the ventral midline once every two days for a total of eight injections over two weeks to promote endometrial hyperplasia and carcinoma-like lesions. Development of endometrial lesions was confirmed by macroscopic observation and histological evaluation of uterine tissue before ASE administration.

After confirmation of endometrial cancer induction, mice were randomly assigned to receive ASE at 0, 50, 100, or 200 mg/200 µL by oral gavage every 2 days for 13 days.

### 2.5. Paraffin Embedding and Sectioning (Histological Slide Preparation)

The liver tissue from each group was separated from the mice, fixed in 70% diethylpyrocarbonate-ethanol, dehydrated, and cleared in 95%, 100% ethanol, xylene. Fixed and dehydrated liver tissues were embedded in paraffin and sectioned at a thickness of 5 µm and 10 µm using a microtome (RMC Boecheler, Tucson, AZ, USA). Sections were mounted on Marienfeld-HistoBond slide glass (Marienfeld, BW, Germany). The slides were dried at 37 °C on a slide-warmer for 48 h.

### 2.6. Hematoxylin and Eosin Staining

The 10 µm thick paraffin-embedded tissue slides were deparaffinized in xylene and rehydrated on 100%, 95%, and 70% ethanol. Sections were stained with hematoxylin and eosin (H&E) (MUTO PURE CHEMICALS Co., Ltd., Tokyo, Japan) for nuclear, cytoplasmic, and extracellular visualization. The slides were dehydrated using the opposite rehydration method and mounted with coverslips.

### 2.7. Alizarin Red S Staining

The 10 µm thick tissue slides were deparaffinized and rehydrated. The slides were stained with 1% Alizarin Red S solution (American MasterTech, Lodi, CA, USA) for 30 min at room temperature. The samples were then rinsed with distilled water to remove excess dye. Stained slides were mounted using a Permount (Fisher Scientific, Fair Lawn, NJ, USA).

### 2.8. Immunohistochemistry

The 5 µm thick slides were deparaffinized, rehydrated, and boiled in 10 mM sodium citrate buffer for antigen retrieval. The slides were incubated in 3% hydrogen peroxide for 5 min and rinsed with 1 × PBS three times. Then they were blocked in 3% Normal Horse Serum for 1 h on room temperature. The primary antibodies were Casp-3 (sc-373730, Santa Cruz Biotechnology, Inc., Dallas,TX, USA), p53 (S15, Cell Signaling Technology, Danvers, MA, USA), LC-3 (ab52628, Abcam, Cambridge, UK), and SQSMT1 (sc-25575, Santa Cruz Biotechnology Inc.). The primary antibodies were detected overnight at 4 °C and secondary antibodies were detected at room temperature for 1 h. Subsequently, the slides were subjected to a chemical reaction in the reactive-substrate (3, 3′-Diaminobenzidine) for a maximum of 10 min. The sections were then rinsed with distilled water and counterstained with hematoxylin. The slides were then dehydrated and mounted using Permount. All steps were rinsed off with 1 × PBS thrice before proceeding to the next step.

### 2.9. Western Blot

Proteins from liver tissue and primary hepatocytes treated with each substance were extracted using Pro-PREPTM protein extraction solution (17081, iNtRON Biotechnology, Seongnam-si, Republic of Korea). The extracted proteins were separated using sodium dodecyl sulfate-polyacrylamide gel electrophoresis and transferred on PVDF membrane. They were blocked subsequently with 3% BSA and primary antibodies were detected at 4 °C overnight. They were then washed with 1 × TBS-T and incubated with a secondary antibody at room temperature for 2 h. The mixture was washed and allowed to react with ECL Substrate () in the dark for 5 min. The samples were exposed and analyzed using the KODAK Image Station 4000R (Carestream Health, Inc., Rochester, NY, USA). Primary antibodies were used for Casp-3 (sc-373730, Santa Cruz Biotechnology Inc.), TNF-R (sc-31349, Santa Cruz Biotechnology Inc.), BCl-xl (2764, Cell signaling Technology, Danvers, MA, USA), TIMP-2 (sc-9905, Santa Cruz Biotechnology Inc.), TIMP-3 (sc-6836, Santa Cruz Biotechnology Inc.), IGF-IR (Insulin-like growth factor I receptor; sc-712, Santa Cruz Biotechnology Inc.), Casp-9 (9508, Cell signaling Technology, Danvers, MA, USA), IL-2 (sc-133118, Santa Cruz Biotechnology Inc.), PI3K (sc-365290, Santa Cruz Biotechnology Inc.), and Akt (sc-5298, Santa Cruz Biotechnology Inc.).

### 2.10. Quantitative Real-Time PCR Analysis

Total RNA was extracted from the tissues using a Total RNA Extraction Kit (SmartGene Soft, Daejeon, Republic of Korea) according to the manufacturer’s protocol. For cDNA synthesis, 1 µg of total RNA was reverse-transcribed using a SuperScript^TM^ III Reverse Transcriptase (18080044, Invitrogen, Thermo Fisher Scientific, Waltham, MA, USA) in a final volume of 20 µL, following the manufacturer’s instructions. Quantitative real-time PCR was performed using SYBR Green PCR Master Mix (SmartGene soft, Daejeon, Korea) on Quanti^TM^ 5 Real-Time PCR System (Applied Biosystems, Thermo Fisher Scientific, Waltham, MA, USA). The PCR cycling conditions were 95 °C for 10 min, followed by 40 cycles at 95 °C for 10 s and 60 °C for 15 s. Primer sequences are listed in Table 1. Relative gene expression levels were calculated using the 2^−ΔΔCt^ method, with GAPDH as the internal control. All reactions were performed in triplicates.

### 2.11. Enzyme-Linked ImmunoSorbent Assay

This experiment was performed to analyze the expression of specific proteins in the liver tissues of mice treated with ASE. The antibodies used were Cytochrome c (A-8) (sc-13156, Santa Cruz Biotechnology, Inc., Dallas, TX, USA), mTOR (sc-517464, Santa Cruz Biotechnology Inc.), PI3K (sc-365290, Santa Cruz Biotechnology Inc.), PCNA (sc-7907, Santa Cruz Biotechnology Inc.), IGF-IR (sc-712, Santa Cruz Biotechnology Inc.), and Akt (sc-5298, Santa Cruz Biotechnology Inc.).

### 2.12. Analysis via ImageJ

To evaluate expression levels on Immuno-analysis results and Western blotting, ImageJ software (Version 1.54f; RRID: SCR_003070; National Institutes of Health, Bethesda, MD, USA) was used for analysis using protocols based on the methods described by Schroeder et al. [10]. The expression sections were localized using the ImageJ analysis tool and quantified using semiautomatic color selection. Each value was analyzed by converting the expression pattern into a percentage based on the value of the negative group.

### 2.13. Statistical Analysis

Statistical analyses were performed using one-way ANOVA followed by Duncan’s multiple range test for post hoc comparisons among groups, with *p* < 0.05 considered statistically significant. All analyses were conducted using IBM SPSS Statistics for Windows, Version 21.0 (IBM Corp., Armonk, NY, USA). Unless otherwise stated, in vitro experiments were performed in three independent biological replicates, each measured in technical triplicate. Data are expressed as mean ± SEM.

## 3. Results

### 3.1. Cytoskeletal Changes and Toxicity Assessment Following Treatment with Natural Substances

**Figure 1:**
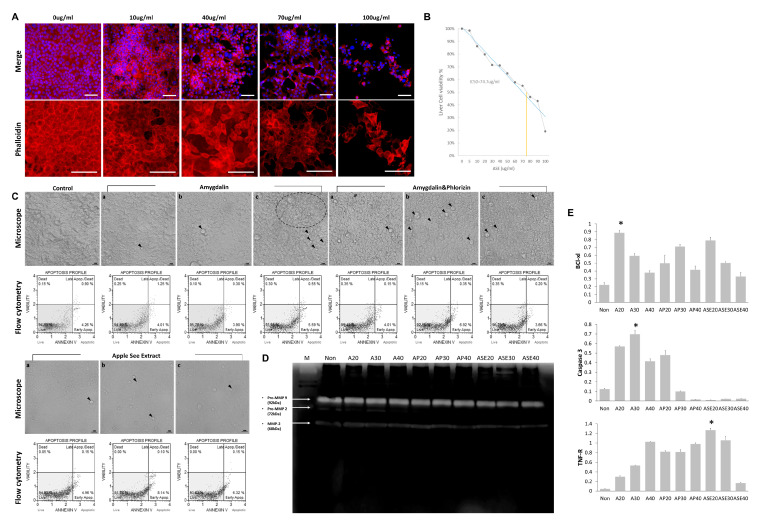
Effects of amygdalin, phlorizin, and apple seed extract (ASE) on hepatocyte viability, apoptosis, and matrix-remodeling markers. The vehicle control (Veh) represents hepatocytes treated with 0.1% ethanol alone. (**A**) Immunofluorescence images of hepatocytes treated with ASE (0–100 μg/mL, 48 h); nuclei were stained with DAPI (blue) and F-actin with phalloidin (red) to assess cytoskeletal disruption and cell loss. (**B**) Cell viability curve of hepatocytes exposed to ASE (0–100 μg/mL, 48 h) measured by CCK-8 assay; viability is expressed as % of non-treated control, and the IC_50_ of ASE is indicated. (**C**) Phase-contrast morphology (upper) and Annexin V flow-cytometry profiles (lower) of hepatocytes treated for 48 h with amygdalin (A20, A30, A40), amygdalin plus phlorizin (AP20, AP30, AP40), or ASE (ASE20, ASE30, ASE40); arrowheads denote multinucleated giant cells, and dot plots show abnormal cell proliferation. a, b, and c indicate the concentrations of the compound: 20 μg/mL, 30 μg/mL, and 40 μg/mL, respectively. (**D**) Gelatin zymography of culture supernatants after 48 h of treatment showing changes in MMP activity; bands of pro/active MMP-2 and MMP-9 are indicated. (**E**) Relative mRNA levels of BCL-xL, Caspase-3, TNF-r, TIMP-2 (Tissue inhibitor of metalloproteinases-2), and TIMP-3 determined by real-time PCR and normalized to the non-treated control. Panels (**A**–**D**) show representative images from three independent experiments. Quantitative data in (**B**,**E**) are presented as mean ± SEM (*n* = 3 independent experiments, each performed in technical triplicate); * *p* < 0.05.

When ASE was treated at 0–100 µg/mL for 48 h, the half-maximal inhibitory concentration (IC50) of the cell viability–concentration curve was calculated to be 74.7 µg/mL (Figure 1). At low concentrations (≤30 µg/mL), polygonal and flat cells predominated, and cortical F-actin was observed uniformly. At intermediate concentrations (≈30–70 µg/mL), decreased stress fiber continuity, discontinuity of the actin ring around the cell periphery, and shrinkage/circularization of individual cells were observed throughout the field of view. At concentrations ≥70 µg/mL (i.e., above the IC50), loss of stress fibers, disruption of intercellular connections, and decreased cell density were observed across a wide field of view, accompanied by fragmentation of actin filaments within the cytoplasm. The state map at the bottom graphically depicts the classification results of cortical maintenance, partial rearrangement, and disassembly according to treatment concentration. The quantitative panel presents the relative values of cell area, circularity, and stress-fiber area ratio (stress-fiber/whole-cell area) calculated from each field of view of the region of interest (Figure 1A,B).

When hepatocytes were treated with amygdalin (A20, A30, A40), amygdalin plus phlorizin (AP20, AP30, AP40), or ASE (ASE20, ASE30, ASE40) for 48 h, phase-contrast images showed that cell shrinkage and apoptotic bodies increased in a dose-dependent manner in the amygdalin and amygdalin plus phlorizin groups, whereas cells exposed to ASE at comparable concentrations largely preserved a smooth surface and higher confluence. In the Annexin V flow-cytometry plots, the proportions of early- and late-apoptotic cells rose sharply in the amygdalin groups, were only partly elevated in the amygdalin plus phlorizin groups, and remained close to control levels in ASE20–40 (Figure 1C). Gelatin zymography of culture supernatants showed several gelatinolytic bands corresponding to pro- and active MMP-2/MMP-9; band intensities tended to increase in some amygdalin and amygdalin plus phlorizin groups, whereas ASE-treated samples were similar to the non-treated control (Figure 1D). Quantitative real-time PCR further revealed that BCL-xL mRNA was higher than control in most treatment groups, while Caspase-3 was significantly upregulated only in A30 and remained low in all amygdalin plus phlorizin and ASE groups. TNF-r expression rose with dose and peaked in AP40 (Figure 1E).

### 3.2. Morphological Changes in Hepatocyte Components and Apoptosis Regulation

**Figure 2:**
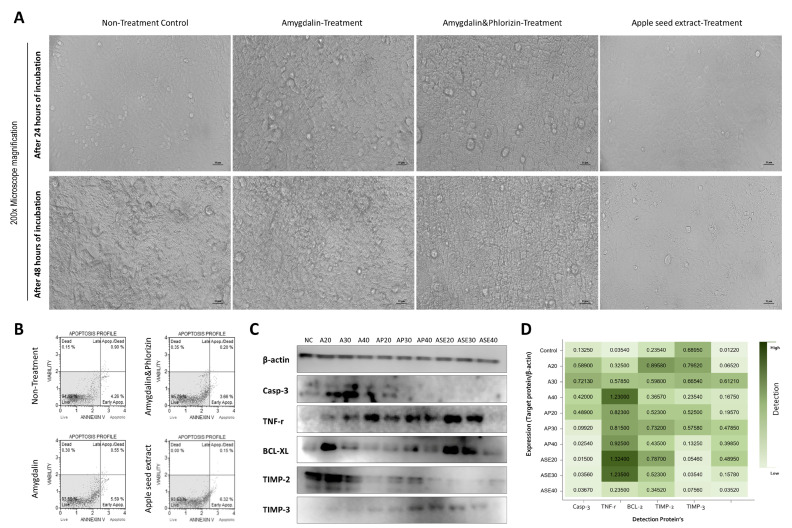
Morphological, apoptotic, and protein-expression changes in hepatocytes treated with amygdalin, amygdalin plus phlorizin, or apple seed extract (ASE). (**A**) Morphological changes were examined by phase-contrast microscopy after exposure to 40 µg/mL amygdalin, amygdalin plus phlorizin, or ASE for 24 and 48 h. Untreated cells formed a flat, confluent monolayer, whereas treated cells showed varying degrees of cell shrinkage and loss of adhesion, which were most prominent in the amygdalin and amygdalin plus phlorizin groups. All images were acquired at 200× magnification. (**B**) Apoptotic profiles in the untreated, amygdalin, amygdalin plus phlorizin, and ASE groups were assessed by Annexin V/propidium iodide staining and flow cytometry. The percentages of viable, early-apoptotic, and late-apoptotic/necrotic cells in each quadrant were used to compare apoptotic responses among treatments. (**C**) Representative Western blots of β-actin, Caspase-3, TNF-r, BCL-xL, TIMP-2, and TIMP-3 in hepatocytes treated for 48 h with amygdalin (A20, A30, A40), amygdalin plus phlorizin (AP20, AP30, AP40), or ASE (ASE20, ASE30, ASE40), and in the untreated control. (**D**) Densitometric analysis of the Western blots shown in (**C**). Band intensities of each target protein were quantified using ImageJ, normalized to β-actin, and presented as heat maps for Caspase-3, TNF-r, BCL-2, TIMP-2, and TIMP-3. All experiments were performed in at least three independent replicates. Panels (**A**–**C**) show representative images from three independent experiments.

At 40 µg/mL, phase-contrast images obtained after 24 and 48 h showed that non-treated hepatocytes maintained a flat, confluent monolayer, whereas all treated groups displayed cell shrinkage and reduced adhesiveness. These changes were most evident in the amygdalin and amygdalin plus phlorizin groups and were comparatively modest in the ASE group (Figure 2A). Annexin V/propidium iodide staining indicated that the proportion of early apoptotic cells in the ASE group (6.3%) was slightly higher than in the amygdalin and amygdalin plus phlorizin groups; however, the total apoptotic fraction (early + late) was greatest in the amygdalin group, followed by amygdalin plus phlorizin and ASE (Figure 2B). At the level of apoptotic signaling, TNF-r expression increased with dose in the amygdalin and amygdalin plus phlorizin series, whereas ASE showed the opposite trend. BCL-xL remained relatively high in the ASE group and decreased as the ASE dose increased. In contrast, Caspase-3 was barely detectable in ASE-treated cells but was strongly expressed in the amygdalin group, despite concomitantly high BCL-xL levels. Differences among treatments were also apparent in the ECM/remodeling profile: densitometric analysis of the Western blots revealed distinct TIMP-2 and TIMP-3 patterns in the amygdalin, amygdalin plus phlorizin, and ASE groups, with ASE tending toward higher TIMP-3 and lower TIMP-2 compared with amygdalin alone (Figure 2C,D).

### 3.3. Histopathological Changes Following ASE Treatment

**Figure 3:**
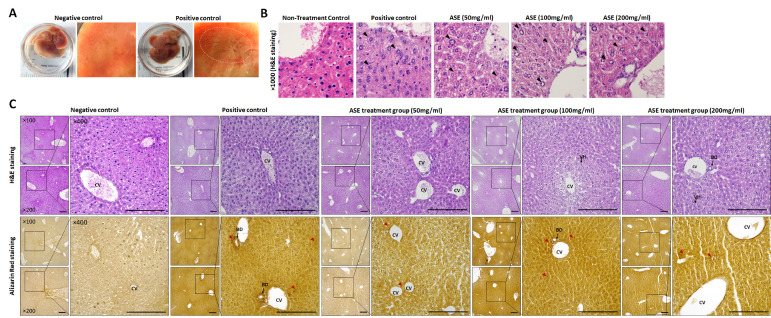
Gross, histological, and calcium-deposition changes in liver tissue from control and ASE-treated tumor-bearing mice. (**A**) Gross appearance of livers from negative-control (NC) and positive-control (PC) mice. NC livers show a smooth, uniformly reddish surface, whereas PC livers display focal discoloration and surface irregularities; the dotted circle indicates a tumor-like lesion on the liver surface. (**B**) High-magnification (×1000) H&E images of peri-lesional regions in NC, PC, and ASE-treated groups (50, 100, and 200 mg/mL). Black arrowheads indicate representative hepatocytes with swelling, cytoplasmic vacuolation, or nuclear atypia in PC and ASE groups, in contrast to the compact hepatic cords in NC. (**C**) Histological analysis of whole-liver sections. H&E staining (upper panels) shows a well-preserved lobular architecture centered on the central vein (CV) in NC, whereas PC livers exhibit reduced cell density and dilated venous sinusoids. In ASE-treated groups (50–200 mg/mL), the lobular field becomes progressively clearer with increasing dose, with fewer and smaller vacuolated hepatocytes and a more linear arrangement of microvessels. Alizarin Red S staining (lower panels) demonstrates increased Ca^2+^ deposition in PC, particularly around CVs and bile ducts (BD; red arrowheads). In ASE50, Ca^2+^ deposits are locally reduced with focal staining near the CV, whereas ASE100–200 show a deposition pattern largely comparable to PC. Panels (**A**–**C**) show representative images from three independent experiments. Scale bar = 100 μm. Abbreviations: NC, negative control; PC, positive control; CV, central vein; BD, bile duct; VH, vacuolated hepatocytes.

On gross examination, the normal control (NC) liver showed a smooth, uniformly reddish-brown surface, whereas the positive control (PC) liver displayed pale, irregular areas consistent with tumor-like lesions on the surface (Figure 3A). At the microscopic level, ×1000 H&E images of perilesional regions revealed densely packed hepatocytes with distinct nuclei in the untreated control, while PC livers contained numerous hepatocytes with cytoplasmic vacuolation, swelling, and nuclear atypia. In the ASE50–200 groups, vacuolated cells were still present but occurred less frequently and less extensively than in the PC group, as indicated by the black arrowheads (Figure 3B).

The NC liver tissue exhibited a radial lobular architecture centered on the central vein (CV), with clearly defined nuclear and cellular boundaries. By contrast, PC liver tissue showed reduced intercellular density and dilated venous sinusoids (microvessels) in multiple fields (Figure 3C, H&E). In the ASE-treated groups, the parenchymal field became progressively clearer with increasing dose, with a gradual decrease in the number and area of vacuolated hepatocytes and a more linear arrangement of microvessels. Alizarin Red S staining demonstrated increased Ca^2+^ deposition in the PC group, particularly around the CV and bile ducts (BDs). In ASE50, the deposition area was reduced in some regions, with focal staining around the CV, whereas in ASE100–200 the overall distribution of Ca^2+^ deposits was largely similar to that observed in the PC group (Figure 3C). Abbreviations: NC, negative control; PC, positive control; CV, central vein; BD, bile duct; VH, vacuolated hepatocytes.

### 3.4. Effect of ASE Treatment on Programmed Cell Death Expression in Mouse Liver Tissue

**Figure 4:**
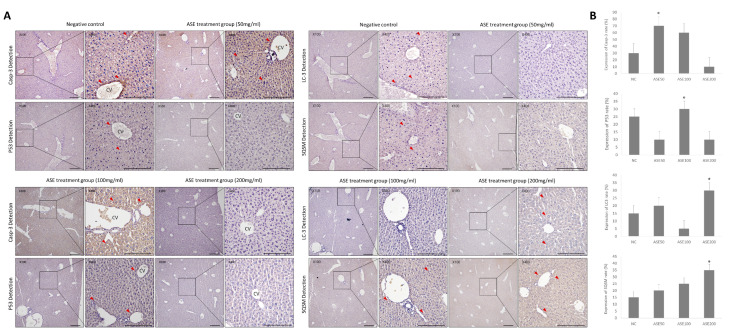
Immunohistochemical analysis of apoptosis- and autophagy-related proteins in the livers of ASE-treated tumor-bearing mice. (**A**) Representative immunohistochemical (IHC) images of Caspase-3, P53, LC3, and SQSTM1 (p62) in liver sections from the normal control (NC) and ASE-treated groups (50, 100, and 200 mg/mL). For each marker, a low-magnification image (×100) around the central vein (CV) and a corresponding high-magnification image (×400) are shown. Red arrowheads indicate prominent immunoreactivity in peri-CV and perivascular areas. (**B**) Quantification of IHC staining. Positive staining areas for Caspase-3, P53, LC3, and SQSTM1 were measured using ImageJ and expressed as the percentage of positive area per field (%) in the NC (Negative control), ASE-treated groups (50, 100, and 200 mg/mL). Panels (**A**) show representative images from three independent experiments. Quantitative data in (**B**) are presented as mean ± SEM (*n* = 3 independent experiments, each performed in technical triplicate); * *p* < 0.05. Scale bar = 100 μm.

In liver tissue, Caspase-3 expression was barely detectable in NC livers but increased markedly in ASE50 and ASE100, and then dropped sharply at ASE200. P53 immunoreactivity was intermediate in NC, lower in ASE50, highest in ASE100, and decreased again in ASE200. For both markers, positive cells were mainly located around the central vein (CV), and in ASE50–ASE100 the stained area extended from the pericentral zone into the surrounding lobular parenchyma, indicating that intermediate ASE doses most strongly activated apoptotic pathways in pericentral hepatocytes (Figure 4A,B). For the autophagy-related markers, LC3 expression showed a slight increase at ASE50, a reduction at ASE100, and the highest signal at ASE200, whereas SQSTM1 (p62) increased stepwise from NC through ASE50 and ASE100 to ASE200. LC3- and SQSTM1-positive cells were not evenly distributed within the lobule but formed repeated clusters around the CV and along sinusoidal spaces, resembling the spatial pattern of the apoptotic markers (Figure 4A,B). The coexistence of enhanced LC3 staining and progressive p62 accumulation at higher ASE doses is more consistent with increased autophagosome formation with limited clearance than with simple acceleration of autophagic flux, suggesting that high-dose ASE is associated with combined apoptotic and autophagy-related stress in pericentral hepatocytes.

### 3.5. Differences in Programmed Cell Death and Survival Signaling

**Figure 5:**
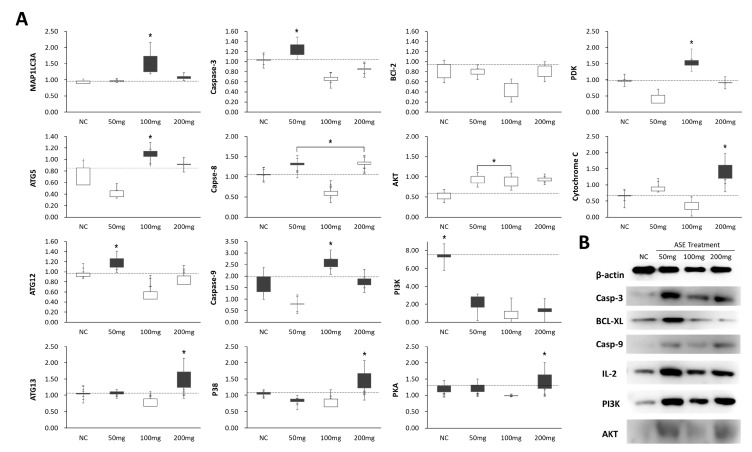
mRNA and protein expression of markers related to apoptosis, survival, and autophagy in liver tissues of ASE-treated tumor-bearing mice. (**A**) Relative mRNA levels of MAP1LC3A, Caspase-3, Bcl-2, PDK, ATG5, Caspase-8, AKT, Cytochrome c, ATG12, Caspase-9, PI3K, ATG13, p38, PKA, and related genes in liver tissues from the normal control (NC) and ASE-treated groups (50, 100, and 200 mg/mL). Expression was quantified by real-time PCR, normalized to GAPDH, and expressed as fold change relative to the untreated control (NC = 1.0; dashed line). Box plots show the distribution of values for individual animals. (**B**) Representative Western blots of β-actin, Caspase-3, BCL-xL, Caspase-9, IL-2, PI3K, and AKT in liver protein extracts from the NC, ASE50, ASE100, and ASE200 groups. All experiments on cell survival and apoptosis factor expression patterns were repeated at least three times. Panels (**B**) show representative images from three independent experiments. Quantitative data in (**A**) are presented as mean ± SEM (*n* = 3 independent experiments, each performed in technical triplicate); * *p* < 0.05.

At the transcript level, several genes showed dose-dependent shifts with ASE treatment. Caspase-3 and PKA mRNA levels were highest at ASE50, Caspase-9 and PDK peaked at ASE100, and BCL-xL expression reached its maximum at ASE200, while proliferating cell nuclear antigen (PCNA) mRNA increased progressively from ASE50 to ASE200 (Figure 5A). Consistent with these transcriptional changes, protein quantification in liver tissue revealed a stepwise increase in Caspase-9 with increasing ASE concentrations, whereas Caspase-3 followed a non-monotonic pattern, showing its highest level at ASE50 and declining at ASE100 and ASE200. In the survival/PI3K–Akt axis, BCL-xL, PI3K, AKT, and IL-2 proteins were most abundant at ASE50 and decreased at higher doses (Figure 5B).

**Figure 6:**
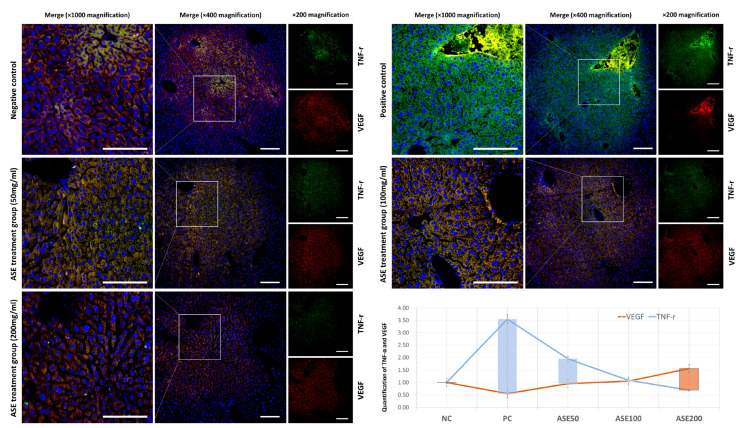
Immunofluorescence analysis of VEGF and TNF-r expression in liver tissues of ASE-treated tumor-bearing mice. Merged immunofluorescence images of VEGF (red), TNF-r (green), and DAPI nuclear staining (blue) in liver sections from the NC, positive control (PC), and ASE-treated groups (50, 100, and 200 mg/mL). For each group, low-magnification (×400) and corresponding high-magnification (×1000) merged images are shown, together with separate TNF-r and VEGF channels (×200). White boxes indicate the regions selected for high-magnification views. The graph shows quantitative analysis of VEGF and TNF-r fluorescence intensities, normalized to the NC group and compared among NC, PC, ASE50, ASE100, and ASE200. Scale bar = 100 μm. Panels show representative images from three independent experiments. Yellow fluorescence indicates co-localization of TNF-r and VEGF. Regions with brighter yellow signals indicate a higher degree of overlap between the two proteins.

Consistent with these results, immunofluorescence staining of VEGF and TNF-R in liver tissue showed clear dose-dependent changes (Figure 6). In the PC group, TNF-R signals were markedly increased around tumor-like regions, whereas VEGF staining was reduced compared with NC. In ASE-treated livers, TNF-r intensity declined gradually from ASE50 to ASE200, while VEGF intensity recovered and, at ASE200, exceeded the level observed in NC. These alterations were most evident in central and perivascular areas, where the number of VEGF-positive hepatocytes increased and TNF-R-positive regions became smaller as the ASE dose increased. Quantitative analysis of fluorescence intensity confirmed that TNF-R reached its maximum in PC and decreased stepwise in the ASE50–ASE200 groups, whereas VEGF showed the opposite tendency, with the highest value at ASE200 (Figure 6).

## 4. Discussion

At subthreshold IC50 doses, most natural product-treated groups exhibited apoptosis rates similar to those of the control group, indicating that overall cytotoxicity remained low. Even so, cell shrinkage and detachment were noted in several treatment conditions, whereas ASE-treated cells showed the fewest morphological changes. In the livers of endometrial cancer-bearing mice, pericentral microvascular dilatation and cytoplasmic vacuolization were more prominent than in the normal group. Both features improved as the ASE dose increased, and the microvascular pattern gradually approached that of the normal control group.

In addition to the architectural changes and modulation of apoptosis–survival markers, Alizarin Red S staining demonstrated focal pericentral calcium deposition in ASE-treated livers. In the context of hepatic pathology, such localized calcium deposits are most consistent with dystrophic calcification, which typically arises in areas of necrosis or long-standing parenchymal injury rather than in otherwise normal liver tissue [11,12,13]. Clinical and experimental reports have shown that hepatic calcification frequently follows ischemic or toxic liver injury and is associated with regions of hepatocellular necrosis and tissue remodeling rather than representing a purely incidental phenomenon [11,13]. In our model, the presence of these pericentral Alizarin-positive foci, together with partial restoration of lobular architecture and changes in apoptosis–survival signaling, suggests that ASE may influence the trajectory of cancer-associated liver injury toward a remodeling phase in which severely damaged zones are sequestered and undergo dystrophic mineralization. Although the precise molecular pathways linking ASE exposure to calcium deposition were not examined here, these findings indicate that the calcium deposits are one component of the hepatic response to ASE under endometrial cancer-related stress.

In parallel, VEGF/TNF-r immunofluorescence showed recovery of VEGF and attenuation of TNF-r signals in peri-central and peri-vascular regions at higher ASE doses, which fits well with the histological picture of partial microvascular normalization.

These molecular and histological patterns are compatible with a rebalancing of survival–death signaling in the damaged liver. TNF-α/TNF-r signaling can drive anti-apoptotic transcription via NF-κB while, at the same time, triggering the death receptor pathway; the final outcome depends on how these two arms are weighted [14]. Bcl-xL suppresses downstream caspase-9/3 activation by preventing mitochondrial cytochrome c release, and hepatocyte studies have consistently pointed to Bcl-xL as a key survival gatekeeper [15,16,17].

In our model, protein analysis showed that caspase-3 peaked at ASE50 and declined at ASE100–200, whereas Bcl-xL, PI3K/Akt, and IL-2 also reached their maximum at ASE50. This pattern suggests a non-monotonic dose window in which pro-survival signals temporarily outweigh executioner caspase activity [2,4] By contrast, some transcripts, particularly caspase-9, showed a shifted maximum at ASE100, diverging from the protein profile. Such a mismatch between mRNA and protein is in line with post-transcriptional regulation or differences in protein stability and will require targeted follow-up [2]. Placed in the broader context of NF-κB/Nrf2, PI3K and Akt, and mitochondrial stress pathways in the liver, these findings point to ASE as a modulator that helps maintain survival signaling while limiting progression to full apoptotic execution under cancer-related stress [2,4]. The dose-dependent decrease in TNF-r and recovery of VEGF expression in ASE-treated livers reinforces this view of a microenvironment that becomes less pro-inflammatory and more supportive of tissue preservation.

Changes in autophagy markers also shape organelle clearance during injury and remodeling. As emphasized by current consensus guidelines, single time-point measurements of LC3 and SQSTM1 (p62) should be interpreted with caution and ideally combined with flux assays using agents such as bafilomycin A1 [18]. In this study, LC3 immunostaining showed a modest increase at ASE50, a dip at ASE100, and a clear rise again at ASE200, whereas p62 accumulated progressively from ASE50 to ASE200, with both markers forming clusters in peri-central regions. Taken at face value, the LC3/p62 directionality is compatible with at least partial recovery of autophagic processing after injury, but formal flux measurements will be needed to determine whether ASE primarily enhances autophagosome turnover or favors autophagosome buildup [18].

The dose-dependent reduction in vacuolization and the partial normalization of the microvasculature after ASE treatment are also in keeping with attenuation of cellular damage and structural recovery. Pericentral, focal Alizarin-positive staining may reflect microcalcification or shifts in calcium-binding matrix components that can accompany liver remodeling in certain injury or metabolic states. At the same time, this pattern should not be overinterpreted as a universal marker of regeneration. Time course experiments combined with vascular and stromal markers such as α-SMA and COL1A1 would help to clarify whether these deposits represent a transient remodeling phase or a more persistent change [19].

Amygdalin has been reported to improve ALT/AST and histological parameters in acetaminophen- and LPS/D-GalN-induced acute liver injury models [3,8]. However, its anticancer efficacy in humans has not been demonstrated, whereas its cyanide-like toxicity is well documented [7]. In this context, the present pattern—attenuation of caspase-3 together with maximal Bcl-xL/PI3K and Akt/IL-2 activity at ASE50 suggests that the polyphenol/antioxidant matrix of ASE produces an apoptosis-modulating profile that is distinct from amygdalin as a single compound [4,5]. Given the dose-dependent nature of amygdalin’s effects and the risk of cyanide-related toxicity, any translational application of ASE will need standardized quantification of its amygdalin content and concurrent monitoring of serum ALT/AST and other safety markers [7].

Importantly, qualitative and quantitative characterization of ASE is necessary to determine the correlation between specific ingredient concentrations and anticancer and hepatoprotective effects. This approach will allow for establishing a realistic efficacy–safety range and identifying the optimal ingredient dosage that maximizes therapeutic efficacy while minimizing cyanide-related risks.

Apple-derived polyphenols such as phlorizin and phloretin have shown hepatoprotective effects in toxin-induced models, mainly through suppression of oxidative and inflammatory stress and support of survival pathways [4,5]. In endometrial cancer settings, modulation of the TNF-α/p53 axis via NF-κB downregulation has been reported, which is broadly in line with our observations on TNF-r/Bcl-xL, caspase-3 signaling [9]. However, differences in cell systems, species, formulations, and dosing regimens make direct comparisons difficult and underline the need for head-to-head studies under harmonized conditions. These in vitro experiments were limited to assessing hepatocyte responses to ASE. Future studies should integrate standardized compositional profiling of ASE with cytotoxicity testing in endometrial cancer cell models to define ingredient-specific dose ranges that maximize anticancer efficacy while preserving hepatoprotection. Additionally, serum liver function markers (ALT/AST) and broader organ toxicity endpoints were not assessed in the current study.

Autophagy was inferred from marker levels rather than directly assessed by flux or ultrastructural approaches. In addition, the discrepancy between Bcl-xL transcript and protein levels remains unexplained and may reflect post-transcriptional regulation or altered protein turnover. Future experiments incorporating proteasome/ubiquitination perturbation, vascular and fibrosis markers, and time-course histology will be important to clarify these mechanisms [17,18].

In summary, ASE reduced caspase-3 dependent apoptosis while preserving Bcl-xL-mediated survival signaling in endometrial cancer-associated liver injury, and was accompanied by less vacuolization and partial normalization of the microvasculature at the tissue level [15,16]. Taken together with the VEGF/TNF-R and autophagy marker profiles, these results support a working model in which antioxidant and anti-inflammatory actions converge on survival pathway preservation at the NF-κB/Nrf2–PI3K–Akt–mitochondrial junction [2,4]. Evaluation of standardized ASE preparations with quantified amygdalin content and systematic safety monitoring will be essential before any translational application can be considered [7]. Collectively, our data indicate that ASE partially mitigates cancer-associated hepatic damage by rebalancing apoptosis–survival signaling and supporting PI3K–Akt-related recovery responses. This pattern implies a dose-sensitive efficacy window. To refine the optimal ingredient dosage range, future studies should combine standardized ASE compositional profiling with endometrial cancer cell cytotoxicity assays and serum-based safety endpoints.

## 5. Conclusions

This study evaluated whether apple seed extract (ASE), utilized in endometrial cancer research, modulates hepatic responses in a mouse model of cancer-related liver injury. Endometrial cancer induction is accompanied by central venous injury and hepatic structural destruction. In this context, specific doses of ASE modulated apoptosis–survival signaling and induced partial remodeling of the liver architecture. These results suggest that appropriate concentrations of ASE compounds may dose-dependently mitigate specific features of cancer-related liver injury and abnormal cell behavior, and may not provide uniform protective effects. However, these results should be interpreted cautiously because apple seeds contain cyanogenic components, and this study did not perform comprehensive systemic toxicity assessments or component-level profiling. Future studies should define clinically meaningful efficacy–safety ranges and establish optimal component doses through rigorous safety assessments, standardized quantification of ASE components, and correlations between specific components and cancer- and liver-related outcome measures.

## Figures and Tables

**Table 1:** Primer sequence.

No.	Gene	Primer (5′ → 3′)	Gene Bank	Product Size (bp)	Tm (°C)
1	***GAPDH***	F: CCCGTTCGACAGACAGCCGTG	NM_001206359.1	238	60
		R: CCGCCTTGACTGTGCCGTGG			
2	***Cytochrome C***	F: GAGGCAAGCATAAGACTGGA	NM_007808.5	133	60
		R: TACTCCATCAGGGTATCCTC			
3	***BAD***	F: GCCCTAGGCTTGAGGAAGTC	NM_007522.3	109	61
		R: CAAACTCTGGGATCTGGAACA			
4	***p53***	F: ACAGTCGGATATCAGCCTCG	NM_011640.4	275	58
		R: TTTTTTGAGAAGGGACAAAAGATG			
5	***Caspase-9***	F: AGTTCCCGGGTGCTGTCTAT	NM_001429869.1	152	60
		R: GCCATGGTCTTTCTGCTCAC			
6	***Caspase-3***	F: CCTCAGAGAGACATTCATGG	NM_009810.3	226	60
		R: GCAGTAGTCGCCTCTGAAGA			
7	***Caspase-8***	F: GCAGAAAGTCTGCCTCATCC	NM_001420041.1	212	60
		R: GGCCTCCATCTATGACCTGA			
8	***BAX***	F: GTGAGCGGCTGCTTGTCT	NM_007527.4	68	61
		R: GGTCCCGAAGTAGGAGAGGA			
9	***p38***	F: TGCCATTCATGGGCACTGAT	NM_001361642.1	402	58
		R: GATTCACAGCCTGAGGGCTT			
10	***BCl-xl***	F: AACATCCCAGCTTCACATAACCCC	NM_001289717.2	94	62
		R: GCGACCCCAGTTTACTCCATCC			
11	***BCl-2***	F: CAGGTATGCACCCAGAGTGA	NM_009741.5	64	60
		R: GTCTCTGAAGACGCTGCTCA			
12	***NF-kB***	F: ACCACTGCTCAGGTCCACTGTC	NM_008689.3	81	63
		R: GCTGTCACTATCCCGGAGTTCA			
13	***PKC***	F: TGGGGTCCTGCTGTATGAGA	NM_011102.5	309	58
		R: TCAAAGTTTTCGCCACTGCG			
14	***VEGF***	F: TTACTGCTGTACCTCCACC	NM_009505.4	189	58
		R: ACAGGACGGCTTGAAGATG			
15	***IGFBP3***	F: AAGCACCTACCTCCCCTCCCAA	NM_008343.2	98	64
		R: TGCTGGGGACAACCTGGCTTTC			
16	***IGF-1***	F: TGTCGTCTTCACATCTCTTCTACCTG	NM_001111276.1	121	62
		R: CCACACACGAACTGAAGAGCGT			
17	***AKT***	F: TGAAAACCTTCTGTGGGACC	NM_007434.4	145	60
		R: TGGTCCTGGTTGTAGAAGGG			
18	***PKA***	F: CAGGAAAGCGCTCCAGATAC	NM_008854.5	229	60
		R: AAGGGAAGGTTGGCGTTACT			
19	***PI3K***	F: AGGAGCGGTACAGCAAAGAA	NM_001077495.2	270	60
		R: GCCGAACACCTTTTTGAGTC			
20	***PDK***	F: CCGGGCCAGGTGGACTTC	NM_172665.5	123	60
		R: GCAATCTTGTCGCAGAAACATAAA			

## Data Availability

The original contributions presented in this study are included in the article. Further inquiries can be directed to the corresponding author(s).
